# Multifocal Electroretinography Changes after UBX1325 (Foselutoclax) Treatment in Neovascular Age-Related Macular Degeneration

**DOI:** 10.3390/jcm13185540

**Published:** 2024-09-19

**Authors:** Nathan Macha, Minzhong Yu, Przemyslaw Sapieha, Sharon Klier, Anirvan Ghosh, Lorraine White, Raj K. Maturi

**Affiliations:** 1 Department of Ophthalmology, Indiana University School of Medicine, Indianapolis, IN 46202, USA; nnmacha@gmail.com; 2 Retina Partners Midwest, and Midwest Eye Institute, Carmel, IN 46290, USA; lorrainew@midwesteye.com; 3 Department of Ophthalmology, University Hospitals, Case Western Reserve University School of Medicine, Cleveland, OH 44106, USA; minzhong.yu@uhhospitals.org; 4 Department of Ophthalmology, University of Montreal, Montreal, QC H3T 1J4, Canada; mike.sapieha@umontreal.ca; 5 UNITY Biotechnology, South San Francisco, CA 94080, USA; sharon.klier@unitybiotechnology.com (S.K.); aniran.ghosh@unitybiotechnology.com (A.G.)

**Keywords:** neovascular age-related macular degeneration, multifocal electroretinography, anti-VEGF, senescence

## Abstract

**Background/Objectives**: The objective of this study was to determine the treatment effect of foselutoclax in neovascular age-related macular degeneration (AMD) by multifocal electroretinography (mfERG) and evaluate mfERG as a potential clinical endpoint in AMD studies. **Methods**: A total of five subjects were included in the study who had active choroidal neovascularization and a history of at least two anti-vascular endothelial growth factor (VEGF) injections in the last 6 months. Subjects received a 50 µL intravitreal injection of foselutoclax at the baseline visit and Weeks 4, 24, and 28 of the study period. **Results**: After foselutoclax treatment, the largest improvement in the mfERG N1-P1 response density occurred at Week 8 as three of five subjects achieved a ≥20% gain. In addition, three of five subjects demonstrated a BCVA improvement of ≥5 ETDRS letters over baseline at Weeks 4, 8, and 24. The mean change in BCVA demonstrated statistical significance in Weeks 4 and 8, showing increases of 5 (*p* = 0.02) and 6.2 (*p* = 0.02) letters, respectively. **Conclusions**: Foselutoclax treatment was shown to have the potential to recover outer retinal function as determined by mfERG and BCVA at approximately Week 8 of treatment.

## 1. Introduction

Intravitreal anti-vascular endothelial growth factor (anti-VEGF) agents have become the standard therapeutic approach for inhibiting angiogenesis in neovascular age-related macular degeneration (nAMD). However, despite adequate anti-VEGF treatment, over 20% of patients with nAMD continue to experience progressive visual deterioration. This ongoing vision loss is often attributed to factors such as subretinal fibrosis, subretinal hemorrhage, and dysfunction of the photoreceptors and the retinal pigment epithelium, as well as the loss of retinal cells in the macular region [1,2].

Recent research has identified the accumulation of senescent cells as a potential key driver of these pathological processes in nAMD. Cellular senescence occurs when cells, following accumulated damage, cease to divide and function properly but remain metabolically active within the tissue. In the context of nAMD, senescent cells can accumulate within the retinal tissues, where they exert detrimental effects by disrupting the blood-retinal barrier, increasing the production of reactive oxygen species and releasing senescence-associated secretory phenotypes (SASPs) [3,4]. The cytokines and other factors secreted as part of the SASP further exacerbate retinal damage by promoting chronic inflammation, tissue degeneration, and angiogenesis, all of which are key features of nAMD [5]. Animal studies have shown that the removal of senescent cells [3] or the suppression of SASP [5] can reduce inflammation and improve the function of remaining retinal cells, suggesting that senolytic therapies may offer a novel approach for treating nAMD.

Foselutoclax (UBX1325, UNITY Biotechnology, South San Francisco, CA, USA), a small-molecule BCL-xL inhibitor, is the first senolytic therapy under clinical development for eye diseases. Foselutoclax selectively induces apoptosis in senescent cells within the retina, thereby potentially mitigating the detrimental effects of cellular senescence. Initial clinical results have shown promise in treating diabetic macular edema, where foselutoclax has been shown to improve retinal endothelial cell function [3]. In the Phase 2 BEHOLD clinical trial, a single injection of foselutoclax led to a statistically significant improvement in mean best-corrected visual acuity (BCVA) by +6.2 letters from baseline to 48 weeks, with 53% of treated patients not requiring any additional treatment through the 48 weeks. Despite these promising findings, there is still limited evidence regarding the impact of foselutoclax on nAMD and the broader influence of senolytic drugs on retinal physiological function.

In this study, we used traditional markers to determine the treatment effect according to BCVA, optical coherence tomography (OCT), and multifocal electroretinography (mfERG), following foselutoclax treatment in nAMD patients. While effective in differentiating retinal disorders, mfERG has rarely been utilized as a clinical endpoint yet, in part due to ambiguity in clinical relevance and variability in mfERG responses longitudinally [6,7]. Thus, this study aims to describe the preliminary findings related to the use of mfERG testing in nAMD and to evaluate its potential as a clinical endpoint. By presenting our observations, we intend to highlight the need for further research to assess the clinical utility of mfERG in future studies.

## 2. Materials and Methods

### 2.1. Study Population and Cohorts

Phase 2 ENVISION was a 48-week, multi-center, double-masked, active-controlled study that enrolled 51 nAMD subjects with active disease despite treatment. The study was conducted from March 2022 to July 2023 and was approved by institutional review boards. All subjects met the following inclusion criteria: at least 50 years of age, at least 2 ani-VEGF injections in the preceding 6-month period, BCVA from 70 to 20 letters, active choroidal neovascularization with the presence of intraretinal or subretinal fluid, and written informed consent. The exclusion criteria included: a concurrent disease in the study eye that could compromise the interpretation of results, infection, or inflammation in either eye in the prior 12 weeks, subretinal hemorrhage with an area of 4 or greater disc area in the study eye, history of vitreous hemorrhage in the study eye in the prior 2 months, and any significant media opacities, such as cataracts, that could interfere with vision of fundus imaging.

Upon a screening visit 4–8 weeks prior to the beginning of the study, each subject received a 2 mg run-in aflibercept intravitreal injection. Subjects were then randomized into 2 cohorts: an experimental cohort and a traditional aflibercept cohort. In the experimental cohort, subjects received a 50 µL foselutoclax intravitreal injection at the baseline visit and at Week 4 during Part A of the study (first 24 weeks) and again at Weeks 24 and 28 during Part B of the study (second 24 weeks). Of the subjects in the study, 5 from the experimental cohort underwent additional mfERG testing at one site. We report on the mfERG findings from this site.

### 2.2. Outcome Measures

In addition to treatment administration, a follow-up evaluation was performed at Weeks 4, 8, 24, 36, and 48 to assess safety, tolerability, BCVA changes, and physiological changes. The Early Treatment Diabetic Retinopathy Study (ETDRS) approximation, letters = 85 − (50 × logarithm of the minimum angle of resolution), was utilized to calculate BCVA, and the same type of VA measurement at each visit was ensured for consistency [8]. Slit lamp examination, intraocular pressure measurement, OCT, and fundus photography assessed anatomical changes and safety profiles during the study period.

For mfERG testing, Diagnosys hardware and related software (Diagnosys LLC, Lowell, MA, USA) were used for recording, following the International Society for Clinical Electrophysiology of Vision guidelines for mfERG [9]. After adequate dilation with tropicamide/phenylephrine eyedrops and topical anesthetic administration of proparacaine eyedrops, a thread-like DTL electrode was placed in the cul-de-sac of each eye, and the patient kept looking at the central fixation point of a liquid crystal display with both eyes open. The Diagnosys system projected a stimulus matrix on the display and consisted of 61 scaled hexagonal elements, which switched between black and white according to an m-sequence during stimulation. The mfERG results were interpreted based on the parameters of the waveforms in the trace array and were assessed for variation spatially at each visit. A custom longitudinal analysis software through Diagnosys was used for analysis. Quantitative analyses of the N1-P1 response densities of the first positive (P1) peak and implicit times of the mfERG traces from summed responses were performed [10,11]. As standard clinical endpoints for nAMD, BCVA and central subfield thickness (CST) were analyzed and compared with the results of mfERG [12,13].

### 2.3. Data Analysis

For all study eyes, changes in BCVA, CST, mfERG N1-P1 response densities, and P1 implicit time from the baseline visit to Weeks 4, 8, 24, 36, and 48 were analyzed. One-tailed paired-sample t-tests were employed. The criteria for statistical significance were set at a *p*-value of <0.05, with all statistical analyses being performed using Microsoft Excel. For mfERG metrics, given their inherent variability and potential sensitivity issues, the absence of statistically significant changes was further interpreted within the context of clinical relevance. Statistical significance was also analyzed in the context of a smaller sample size for this preliminary study.

### 2.4. Ethics Statement

The clinical studies were conducted at multiple study sites in compliance with the Declaration of Helsinki, US Code 21 of Federal Regulations, and the Harmonized Tripartite Guidelines for Good Clinical Practice (1996) and were reviewed and approved by the appropriate Ethics Committees or institutional review boards (Advarra with an approval date of 20 May 2021., single IRB used, 100 Merriweather Dr., Suite 600, Columbia, MD 21044). Informed consent was obtained from all study participants. Patients were not compensated for trial participation. ClinicalTrials.gov ID: NCT05275205.

## 3. Results

This study included five subjects in the experimental cohort of the Phase 2 ENVISION study. Demographic information and baseline clinical features, including BCVA, CST, total N1-P1 response density, and P1 implicit time, are summarized in Table 1.

**Figure 1:**
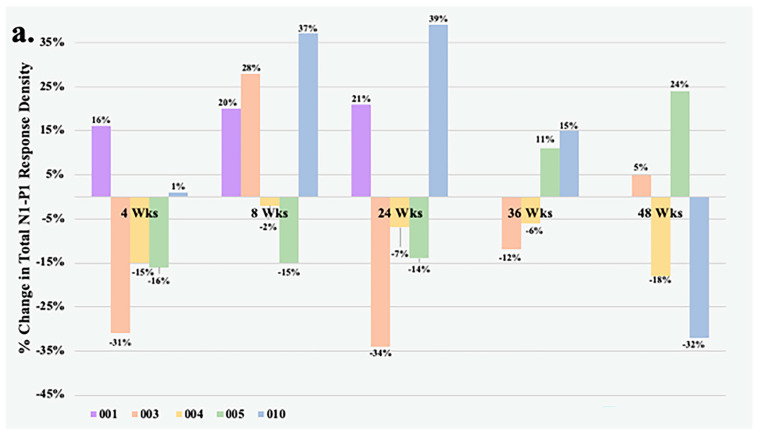

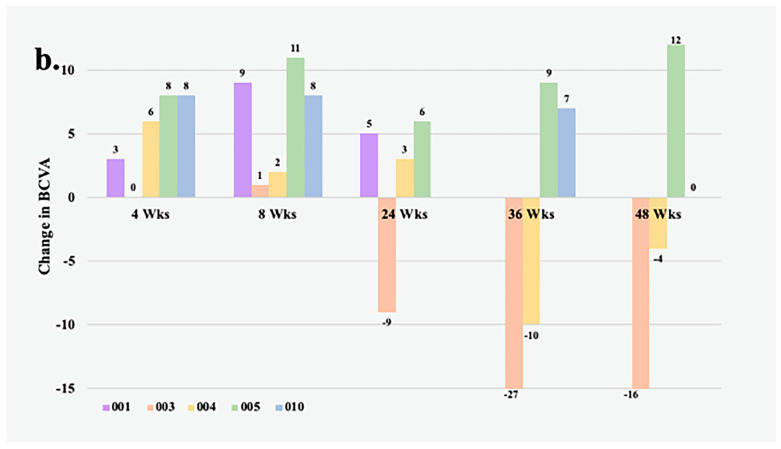
(**a**) Percentage change from baseline in the total N1-P1 response density and (**b**) changes from baseline in BCVA at each visit of all study eyes. Subject 001 was unable to undergo testing at Weeks 36 and 48. Subjects 003 and 004 both received rescue aflibercept treatments at Weeks 24 and 36.

Figure 1 details the change in total N1-P1 response density and BCVA over time of all study eyes that underwent mfERG testing. At Week 4, all but one eye had less than a 20% change in the total N1-P1 response density from baseline. The largest improvements in response from the foselutoclax treatment during the study period occurred at Week 8, as three of five subjects achieved a ≥20% gain in total N1-P1 response density, including Subjects 001 (+20%), 003 (+26%), and 010 (+37%). Subjects 001 and 010 remained at a ≥20% gain in total N1-P1 response density at Week 24, before all study eyes returned to normal range of change in total N1-P1 response density at Week 36. One foselutoclax-treated eye, Subject 005, achieved a ≥20% gain in total N1-P1 response density at Week 48 (+24%), while another, Subject 010, demonstrated a ≥20% loss in total N1-P1 response density (−32%). The total N1-P1 response density did not reach statistical significance at any visit but did have a noteworthy gain of 14% at Week 8 compared to baseline.

Within the foselutoclax-treated cohort, three of five subjects demonstrated a BCVA of ≥5 ETDRS letters over baseline at Weeks 4, 8, and 24, which is equivalent to a single line of visual acuity. Mean change in BCVA demonstrated statistical significance at Weeks 4 and 8, showing increases of 5 (*p* = 0.02) and 6.2 (*p* = 0.02) ETDRS letters, respectively (Table 2). Additionally, two of five subjects achieved a BCVA gain of ≥5 ETDRS letters over baseline at Weeks 36 and 48. A strong correlation between N1-P1 response density and BCVA was observed in some subjects, best exemplified by Subject 001 (r = 0.81) and Subject 010 (r = 0.64). However, visual improvements did not consistently align with correlated total N1-P1 response density increases, as evidenced by Subject 003 (r = 0.15) and Subject 004 (r = −0.05). It should be noted that Subjects 003 and 004 required rescue treatments of aflibercept at Weeks 24 and 36 due to increased edema, which was associated with both decreased BCVA and total N1-P1 response density.

It is important to highlight that mean CST consistently rose at each subsequent visit; however, none of these changes required statistical significance. Moreover, the secondary mfERG parameter, mean P1 implicit time, did not display a significant change throughout the study period.

**Figure 2:**
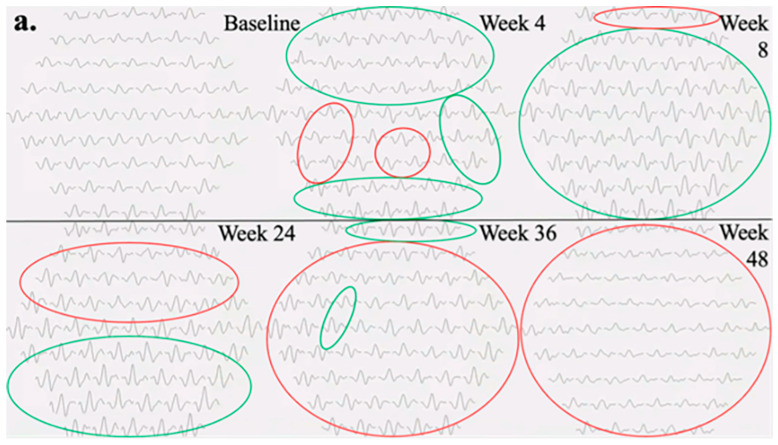

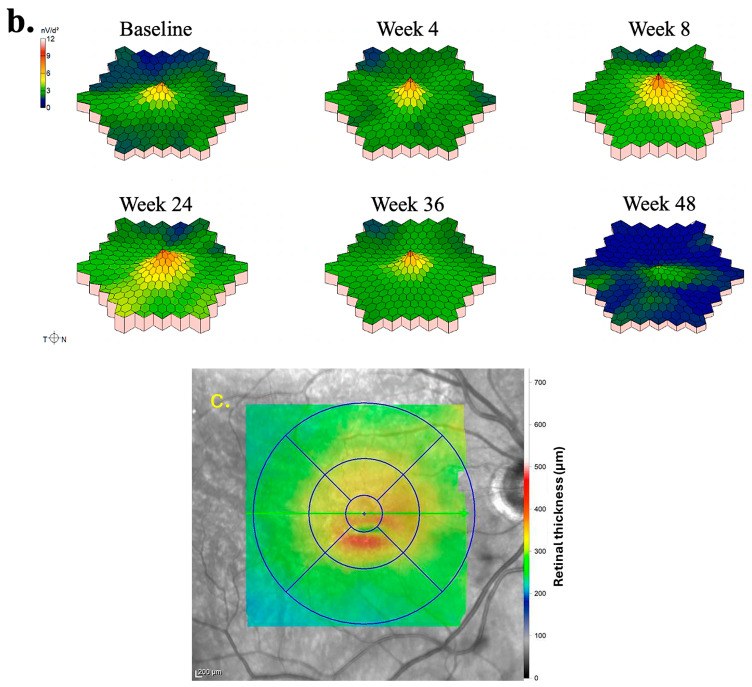
Testing results of Subject 010, including (**a**) mfERG trace arrays at each visit, (**b**) mfERG 3D topographical distributions at each visit, and (**c**) baseline OCT retinal thickness heat map overlayed on fundus imaging. In trace arrays, green circles indicate regions of increased N1-P1 response densities from baseline, and red circles indicate regions of decreased N1-P1 response densities from baseline. mfERG plots show the response density increases to the maximum at Week 8 and reduces from Weeks 24 to 48.

The subsequent portion of results details the mfERG findings of two eyes treated with foselutoclax that demonstrated the most improvement, particularly at Week 8 of the study period. The mfERG findings for the right eye of Subject 010 are summarized in Figure 2. Examination of individual mfERG traces revealed large regions of improvement at Week 4, albeit accompanied by localized inferotemporal regression (Figure 2a). By Week 8, nearly all traces showed improved responses, a trend correlating with the administration of foselutoclax at baseline and Week 4. Subsequent to Week 8, widespread regression emerged, notably affecting the superior retina at Week 24 and virtually all mfERG traces at Weeks 36 and 48. The 3D topographic map at each visit supplemented the findings derived from trace arrays (Figure 2b). The 3D topographic map at Week 4 demonstrated the restoration of the central peak, a feature maintained through Week 36. The central peak regressed moderately at Week 36 before disappearing at Week 48. The region of edema was primarily localized to the inferior parafovea at baseline (Figure 2c). This region exhibited no evident correlation with alterations in trace arrays as these traces often changed synchronously with neighboring traces, with the exception of Week 4.

The quantitative outcome measures of Subject 010 are summarized in Table 3. After a single injection of foselutoclax, the eye achieved a BCVA gain of eight ETDRS letters, accompanied by enhancements in both total N1-P1 response density and P1 implicit time. At Week 8, a notable increase in total N1-P1 response density by 6441 nV/deg^2^ and a decrease in P1 implicit time by −1.8 ms from baseline were observed, while BCVA remained stable. Despite a sustained total N1-P1 response density, CST experienced a marked increase at Week 24 of 50 µm from baseline. By Week 48, BCVA and P1 implicit time reverted to baseline levels, while N1-P response density regressed below baseline.

**Figure 3:**
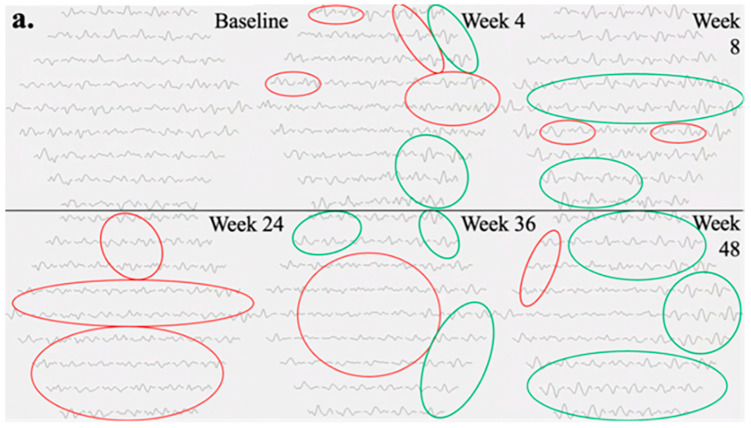

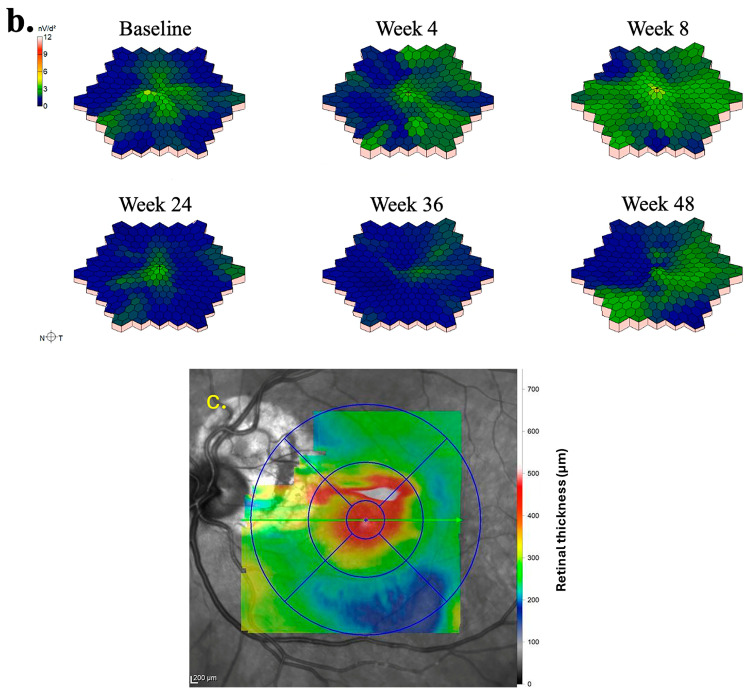
Testing results of Subject 003, including (**a**) mfERG trace arrays at each visit, (**b**) mfERG 3D topographical distributions at each visit, and (**c**) baseline OCT retinal thickness heat map overlayed on fundus imaging. In trace arrays, green circles indicate regions of increased N1-P1 response densities from baseline, and red circles indicate regions of decreased N1-P1 response densities from baseline. If an individual waveform was identified as noise, it was excluded from assessment for fluctuations in amplitude. mfERG plots show the response densities increase to the maximum at Week 8 and reduce from Weeks 24 to 48.

The mfERG findings pertaining to the left eye of Subject 003 are summarized in Figure 3. Temporal regions of individual hexagon responses revealed both improvements and regressions at Week 4 with moderate noise in rings 1 and 2, attributable to persistent central edema (Figure 3a). At Week 8, numerous regions exhibited improvement, including the superior and inferior macular. As mentioned previously, this subject experienced severe cystic edema following Week 8, necessitating the usage of rescue aflibercept treatments at Weeks 24 and 36. Accordingly, responses at Week 24 declined in similar regions to those that improved at Week 8. By Week 36, the cystic edema became more widespread, leading to predominantly low waveforms. Following rescue treatments, superior and inferior mfERG responses rebounded as the edema improved, becoming primarily localized to foveal regions at Week 48. Three-dimensional topographies demonstrated a similar trend to trace arrays with improved responses up to Week 8, as well as severely regressed topographies at Weeks 24 and 36 (Figure 3b). At baseline, the foveal and parafoveal regions exhibited the most significant edema, which consistently correlated with noise throughout subsequent visits (Figure 3c).

Table 4 summarizes the quantitative outcome measures for Subject 003. At Week 4, compared to the baseline, the total N1-P1 response density decreased by 3038 nV/deg^2^, while the central subfield thickness (CST) increased by 36 µm. By Week 8, the N1-P1 response density significantly improved, exceeding baseline levels by 2782 nV/deg^2^, and the best-corrected visual acuity (BCVA) remained stable. However, with the onset of increased cystic edema, both BCVA and N1-P1 response density deteriorated by Weeks 24 and 36. Although BCVA was relatively unchanged at Week 24, CST rose significantly by 354 µm at Week 36 compared to baseline. At Week 48, the N1-P1 response density slightly exceeded baseline levels, and BCVA improved by 12 ETDRS letters from Week 36. The P1 implicit time did not show a strong correlation with anatomical changes or other quantitative measures in these subjects.

## 4. Discussion

The mfERG testing in this study provided an objective and accurate evaluation of retinal physiological functioning in nAMD. The total N1-P1 response density tended to correlate moderately with anatomical changes in eyes, despite not consistently correlating with BCVA improvement in certain subjects. Across all subjects and visits, waveforms demonstrated minimal noise overall, affirming the reliability of the testing in this study. In regions of severe edema in the macula, low but noisy waveforms occurred, which may have impacted 3D topographical distributions and total N1-P1 response density values. In these cases, trace arrays were evaluated to confirm findings.

The mfERG results and other metrics indicated an improvement in function in most participants with nAMD assessed in this study who were treated with foselutoclax. Participants enrolled had the active disease despite treatment, and the most notable improvements occurred at Week 8 of the study period, following the administration of foselutoclax injections at baseline and Week 4. At this visit, an increased average total N1-P1 response density and a statistically significant BCVA improvement were observed. Increased response densities did not appear to be localized to the region of macular edema but rather widespread across most traces in the stimulus matrix. By the end of the study, most quantitative parameters, including total N1-P1 response density and BCVA, reverted to near baseline, while CST appeared to increase slightly during the study period. This suggests foselutoclax treatment may require a higher dosing frequency or rather be used in combination with an anti-VEGF agent. Moreover, the possibility of combining foselutoclax with anti-VEGF therapy offers an intriguing avenue for enhancing treatment durability. Existing literature on combination therapies for nAMD suggests that such approaches could synergistically address multiple pathways involved in the disease, potentially leading to more sustained improvements in retinal function and visual acuity [14]. By considering these factors, our study contributes to the ongoing discussion on optimizing treatment strategies for nAMD, highlighting the need for further research into dosing schedules and combination therapies to maximize therapeutic efficacy.

The primary objective of this study was to evaluate the effectiveness of mfERG as a clinical endpoint in assessing retinal function in patients with nAMD treated with foselutoclax. However, the interpretation of our findings is limited by several factors, particularly the small number of participants and the variability in the mfERG response density across individuals. These constraints raise questions about the reliability of mfERG as a definitive clinical endpoint in this context.

One notable limitation is the limited sample size, which restricts the statistical power of the study and may contribute to the variability in the mfERG outcomes observed. Statistical significance in this study was used to assess the preliminary results of these subjects. In small cohorts, individual variability in disease severity, retinal morphology, and response to treatment can disproportionately affect the results, leading to inconsistencies in mfERG measurements. Additionally, nAMD is a highly heterogeneous disease, with varying degrees of photoreceptor and retinal pigment epithelium damage, which can further complicate the interpretation of mfERG data. This inherent variability in disease presentation may make it challenging to establish uniform mfERG response patterns across patients, potentially limiting the utility of mfERG as a robust endpoint in small-scale studies.

Despite these challenges, mfERG remains a valuable tool for assessing localized retinal function, particularly in the macular region affected by nAMD. A key finding of this study was, of the subjects that showed early mfERG response improvement by Week 8 (Subjects 001, 003, and 010), only one required rescue treatment during the study. This indicates a potential for early prognostication with mfERG testing. Our findings align with previous research that has demonstrated both the potential and limitations of mfERG in nAMD [10,15,16,17]. Studies utilizing mfERG have reported mixed results regarding its sensitivity and specificity in detecting functional changes following treatment. For instance, some studies have shown significant improvements in mfERG response density following anti-VEGF therapy, while others have reported more modest or inconsistent findings, particularly in patients with advanced disease stages or extensive retinal damage. These variations suggest that, while mfERG can capture functional changes in the retina, its reliability as an endpoint may depend on the specific characteristics of the patient population and the stage of nAMD being studied.

In light of these observations, future studies with larger sample sizes and more homogeneous patient populations are needed to better define the role of mfERG as a clinical endpoint in nAMD. Additionally, further research should explore the integration of mfERG with other functional and anatomical assessments, such as BCVA and OCT, to provide a more comprehensive evaluation of treatment efficacy. By combining multiple endpoints, it may be possible to reduce variability and enhance the reliability of mfERG as a tool for monitoring retinal function in nAMD.

To conclude, foselutoclax treatment led to an improvement in retinal physiological functioning in patients with nAMD, as assessed by mfERG testing, and an improvement in BCVA. The longitudinal assessment of individual patients provided important temporal information, suggesting an effect of foselutoclax at 8 weeks post-treatment initiation. This study also suggested that the total N1-P1 response density of mfERG testing, in combination with trace array evaluation, may serve as an independent clinical endpoint measurement to supplement BCVA and CST in nAMD studies. While our study highlights the potential of mfERG as a clinical endpoint, the limitations related to sample size and patient heterogeneity must be acknowledged. These constraints underscore the need for further research to establish the most reliable methods for assessing retinal function in nAMD, particularly in the context of novel therapeutic approaches such as foselutoclax.

## Figures and Tables

**Table 1:** Subject Demographics and Baseline Clinical Features.

Subject Number	Age	Sex	Baseline BCVA (Letters)	Baseline CST (µm)	Baseline P1 Response Density (nV/deg^2^)	Baseline P1 Implicit Time (ms)
001	79	Male	25	423	11,762	35.4
003	78	Male	52	454	9940	34.5
004	86	Female	63	451	11,597	39.1
005	93	Female	60	308	8327	38.7
010	91	Male	72	311	17,230	34

**Table 2:** Mean changes in BCVA, CST, Total N1-P1 Response Density, and total P1 Implicit Time in the Foselutoclax-treated Cohort. “*” indicates the value reached statistical significance (*p* < 0.05).

	Week 4	Week 8	Week 24	Week 36	Week 48
BCVA (letters)	5 *	6.2 *	2	−5.25	−2
CST (µm)	5%	10%	10%	31%	33%
N1-P1 Response Density (nV/deg^2^)	−9%	14%	1%	2%	−5%
P1 Implicit Time (ms)	−0.8	−0.62	0.3	−0.75	0.23

**Table 3:** BCVA CST, Total N1-P1 Response Density, and Total P1 Latency Results of Subject 010.

	Baseline	Week 4	Week 8	Week 24	Week 36	Week 48
BCVA (letters)	72	80	80	77	79	72
CST (µm)	311	317	331	361	356	328
N1-P1 Response Density (nV/deg^2^)	17,230	17,332	23,671	24,024	19,772	11,642
P1 Implicit Time (ms)	34	33	32.1	33.7	32.7	34.1

**Table 4:** BCVA CST, Total N1-P1 Response Density, and Total P1 Latency Results of Subject 003.

	Baseline	Week 4	Week 8	Week 24	Week 36	Week 48
BCVA (letters)	52	52	53	43	25	36
CST (µm)	454	490	480	494	797	768
N1-P1 Response Density (nV/deg^2^)	9940	6902	12,722	6518	8795	10,471
P1 Implicit Time (ms)	34.5	33	34.8	34.5	33.3	34.4

## Data Availability

Data are contained within the article.
